# Does a policy that requires adherence to a regular primary care physician improve the actual adherence of patients?

**DOI:** 10.1186/s13584-021-00475-9

**Published:** 2021-08-25

**Authors:** A. Golan-Cohen, G. Blumberg, E. Merzon, E. Kitai, Y. Fogelman, A. Shipotovsky, S. Vinker

**Affiliations:** 1 Leumit Health Services, 23 Shprinzak St, Tel Aviv, Israel; 2 Tel Aviv University Faculty of Medicine, 6927901 Tel Aviv, Israel; 3 Technion Faculty of Medicine, Haifa, Israel

**Keywords:** Continuity of care, Personal physician, Adherence, Usual provider of care

## Abstract

**Background:**

Continuity of care by the same personal physician is a key factor in an effective and efficient health care system. Studies that support the association between high adherence and better outcomes were done in settings where allocation to the same physician was a long-term policy.

**Objectives:**

To evaluate the influence that changing organizational policy from the free choice of a primary care physician to a mandatory continuity of care by the same physician has on adherence to a personal physician.

**Methods:**

A cross-sectional study based on electronic databases; comparison of adherence and demographic characteristics (sex, age, and socio-economic status) of 208,286 Leumit enrollees who met the inclusion criteria, according to change in the adherence to a personal physician. To evaluate adherence, we used the Usual Provider of Care (UPC) index, which measures the number of visits made to the personal doctor out of the total primary care physician visits over the same period. The patients were divided into groups according to their UPC level.

**Results:**

The data shows that 54.5% of the patients were high adherers even before the organizational change; these rates are similar to those published by various organizations worldwide, years after mandating continuity of care by the same physician.

In the year following the intervention, only 34.5% of the patients changed the level of their adherence group. Of these, 64% made a shift to a higher adherence group.

Before the intervention, the high adherers were older (mean age 57.8 vs. 49.3 years in the low adherers group) and from a higher SES (mean SES status 9.32 vs. 8.71). After the intervention, a higher proportion of older patients and patients from a higher SES changed their adherence to a higher group.

Sex distribution was similar over all the adherence level groups and did not change after the intervention.

**Conclusions and policy implications:**

A policy change that encouraged adherence to an allocated primary care physician managed to improve adherence only in specific groups.

Health organizations need to examine the potential for change and the groups they want to influence and direct their investment wisely.

**Trial registration:**

retrospectively registered.

## Background

Continuity of care is a basic tenet of good medical practice [1]. It forms an infrastructure that combines data continuity, continuity of treatment among the different sectors, and better accessibility to care [2, 3]. A deeper understanding of the patient over time helps both patient and physician develop a relationship of trust [4–7], enabling better adherence due to more familiarity.

Continuity of care in primary care is essential, and several literature reviews have shown that quality of care is improved when patients stay with one primary care doctor over time [8–10]. Among the outcomes that are influenced are better adherence to diagnostic tests and treatments offered as preventive medicine [11], decreased hospitalizations [11–14], reduced over-use of healthcare resources [ 15–18], better-controlled chronic diseases (including psychiatric conditions) [ 19–24], reduced mortality [9, 16, 25] and improved communication and trust between the physician and the patient [5–7]. Other studies support the findings that continuity of care contributes to increased patient satisfaction [ 21, 26–29], and physician satisfaction [ 30].

However, the methodology of most of these studies is equivocal. One review, which attempted to use studies with better methodology, only found that continuity of care influences the decreasing use of healthcare resources. No support was found for the improvement of patient outcomes [ 30].

Healthcare organizations are motivated to aspire to optimal quality of care. Efforts span from a continuity of care as contributing to better health, economics, and service to patients, to invest in creating mechanisms that encourage the use of a personal primary care physician. The latter requires administrative tools that monitor and guide the interactions between physician and patient using incentives and blocks that encourage continuity of care, for both the physician and the patient.

Good adherence is usually defined as at least 70% of physician visits to the personal physician over time [7, 18, 19, 31]. Studies from healthcare organizations worldwide that use tools to encourage the bond between patient and personal physician demonstrate that only 50–60% of the population show good adherence. The fact that not all patients adhere to their personal physician, despite the healthcare organization’s policies, raises the question of these rules’ influence on patient behavior. Considering that almost all the studies have been retrospective, no cause-effect relationship could be established. For example, we cannot conclude that there is a correlation between adherence and better trust in the personal physician; there is a possibility that a patient who takes better care of his health, in general, has inherent characteristics that should be considered as confounding variables [9, 10, 19]. Besides, due to the scarcity of data, it is acceptable to invest in mechanisms that increase adherence to a personal physician for all patients. However, it would probably be enough to involve either targeted populations with lower adherence or those where it is reasonable to assume that such an intervention would significantly improve outcomes. We were unable to find a study that evaluated the influence of adherence on patients who previously were able to move between primary care doctors. Thus, it is difficult to answer this question.

Leumit Health Services is one of four nationwide health maintenance organizations in Israel, serving a patient population of about 720,000. In 2014 an organizational change was instituted, which allowed us to evaluate adherence to a personal physician. Until 2014, our patients were free to visit any primary care physician they chose at any available appointment. In 2014 the system changed, and each patient was allocated to a personal primary care physician. The personal physician was defined as the one that the patient visited most in the past year. The general rule was that appointments could be scheduled only to the personal physician unless the personal physician is not available in the next two working days and, according to the patient’s judgment, there is a good reason for an earlier appointment. The new model was gradually implemented over a few months.

In this study, we evaluated the influence of this organizational change on adherence levels to a personal physician when no other significant changes were made in our healthcare system.

## Methods

### Study design

Population-based cross-sectional study.

### Study period

June 2013–June 2014, the period before implementation of a personal physician model.

July 2014 – December 2014, implementation period.

January 2015–December 2015, the period after implementation of a personal physician model.

### Study population

#### Inclusion criteria

Patients who were Leumit enrollees throughout the study period of June 2013 to December 2015, were 20 years old or older at the beginning of the study (age at baseline) and had at least three appointments with a primary care physician in the period before and the period after the implementation.

#### Exclusion criteria

- Leumit enrollees who were younger than 20 years (*N* = 262,843). We assumed that most of them did not choose by themselves the doctor they visited.
- Patients who changed address during the study period or patients allocated to clinics that did not have the same primary care doctors during the whole study period (*N* = 207,604).
- Patients with one of the five “serious diseases” as defined in the National Health Law (patients on dialysis, thalassemia major, AIDS, hemophilia, Gaucher) (*N* = 6380).
- Patients with a diagnosis of active malignant disease (*N* = 29,615).
- Patients allocated to Home Care Units (*N* = 2317).

### Population size

Two hundred eight thousand two hundred eighty-six patients met the inclusion criteria.

### Index of continuity of care

Many studies have proposed indices. Some are based on defining the personal physician in advance; others looked at patient decisions in action. Studies that looked at the various methods to see if the data obtained was similar, found that specificity and sensitivity values were similar [7, 9, 10, 14, 28, 32]. Among the indices tested, we chose Usual Provider of Care (UPC), which expresses the ratio of the number of visits made to the personal doctor to the number of total visits over the same period. It is calculated by dividing the number of visits to the same doctor by the number of all primary care doctor visits over the same period. The doctor whom the patient visited the most over this period was considered the personal physician. The UPC is not dependent on naming the personal physician initially and is thus suited to our study [31]. The patients were divided into three groups according to the use of UPC: very low adherence (UPC ≤ 50%), middle adherence (UPC > 50 and < 70%), and high adherence (UPC > 70%). For further analysis, we merged the very low and middle adherence groups to one group, defined as low.

### Other measurements

Sex, age, and socio-economic status (SES) as a continuous variable reflecting levels according to the Israeli Central Bureau of Statistics where 1 is the lowest level and 20 the highest [32]. Low-middle SES equals socio-economic groups 1–10, and middle-high SES equals groups 11–20.

### Statistical analysis

Continuous demographic characteristics, such as age at baseline and SES, are presented as the mean and 95% confidence interval. Categorical data are shown in counts and percentages.

All subjects were categorized into groups according to their UPC before and after the change. The association between the adherence level to a personal physician before and after the change was evaluated using each group’s population characteristics and its correlation with the UPC category. Initially, chi-square tests and independent t-tests were employed for categorical and continuous variables, respectively. The one-way ANOVA test was initially applied to the data, and the Bonferroni hoc was used to identify the difference between the categories (*p* > 0.05).

## Results

Table 1 shows the distribution of variables according to patient adherence rates before and after the intervention. The results show that 54.5% (CI 95%, 54.9–55.1) of the patients were high adherers before the organizational change. In the year following the intervention, all groups showed significantly increased adherence to their personal physician, while the number of high adherers increased by 9.5% (*p* < 0.001).

**Table 1:** Patient characteristics by UPC categories (Low, Middle, High)

UPC category	Total Patients N (%)			Age at baseline Mean (CI)			Socio-economic status Mean (CI)			Female sex N (%)		
	Before	After	***p***-value	Before	After	***p***-value	Before	After	***p***-value	Before	After	***p***-value
**Total**	208,286 (100%)	208,286 (100%)		**54.9 (54.9–55.1)**	**54.9 (54.9–55.1)**		**9.16 (9.14–9.16)**	**9.16 (9.14–9.16)**		**119,968 (57.6%)**	119,968 57.6%)	
**Low** **(<=50%)**	31,656 (15.2%)	21,238 (10.2%)	< 0.001	49.3 (49.1–49.5)	48.7 (48.5–48.9)	=0.041	8.71 (8.62–8.77)	8.34 (8.28–8.39)	=0.006	18,664 (59%)	12,398 (58%)	=0.046
**Middle** **(> 50 and < 70%)**	63,072 (30.3%)	53,669 (25.7%)	< 0.001	52.6 (52.5–52.7)	51.6 (51.4–51.8)	=0.012	9.07 (9.04–9.10)	8.82 (8.79–8.9)	< 0.001	36,274 (57.5%)	31,083 (57.9%)	=0.042
**High** **(=> 70%)**	113,559 (54.5%)	133,379 (64.0%)	< 0.001	57.8 (57.7–57.9)	57.3 (57.2–57.4)	=0.036	9.32 (9.30–9.34)	9.42 (9.40–9.44)	=0.014	65,030 (57.26%)	764,87 (57.35%	=0.0486

Before the intervention, the high adherers were older than the other groups (mean age 57.8 years (CI 95%, 54.9–55.1) vs. 49.3 years (CI 95%, 49.1–49.5) in the low adherers and 52.6 years (CI 95%, 52.5–52.7) in the middle adherence group (*P* < 0.001)).

**Fig. 1:**
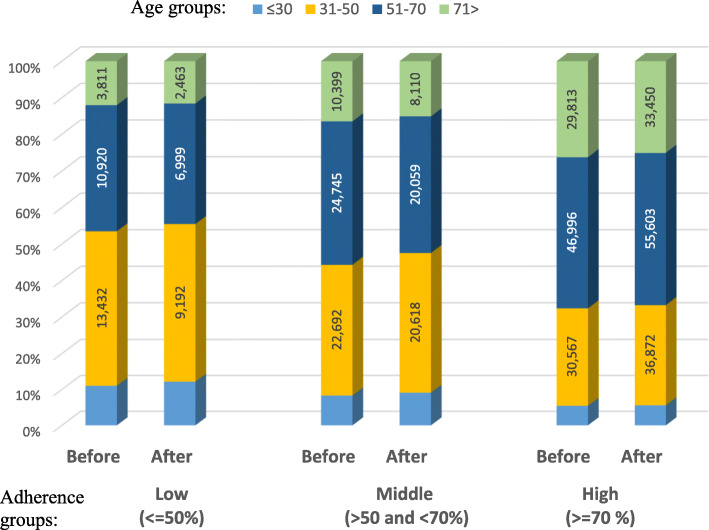
The categorical baseline age distribution in each adherence group, before and after the intervention

Figure 1 represents the population shift according to the age groups after the intervention. The results show that after the organizational change, the higher proportion of patients in the low and the middle adherence groups that changed their adherence to a higher one were older. Thus the mean age in each group after the intervention was significantly lower (*P* < 0.05 for all the groups).

The same pattern of change was found when we evaluated the socio-economic status in the different adherence groups. Before the intervention, the high adherers were from a higher SES than the other groups (mean SES status 9.32 (CI 95%, 9.30–9.34) vs. 8.71 (CI 95%, 8.62–8.77) in the very low adherers and 9.07 (CI 95%, 9.04–9.10) in the middle adherence group (*P* < 0.05)). After the intervention, there was a shift of patients in the higher SES status. They moved from the very low and in the middle adherence groups to the higher adherence group. Thus, the mean SES in these groups became significantly lower (*P* < 0.05) after the intervention. In contrast, the mean SES in the high adherence group increased to 9.42 (CI 95%, 9.40–9.44) [*P* < 0.05]. Sex distribution was similar over all the adherence level groups and did not change after the intervention.

Table 2 shows the characteristics of the patients divided into four groups, according to adherence level before and after the intervention. Each group was also divided into sub-groups by age, socio-economic status, and sex.

**Table 2:** Patient characteristics by groups of adherence, according to adherence level before and after the intervention

Variables	Total Patients 208,286(100%)	Low-Low 48,873(23.5%)	Low-High 45,855(22.0%)	High-Low 26,034(12.5%)	High-High 87,524(42.0%)	***P***-value
**Age at baseline Mean (CI)**	55.0 (54.9–55.0)	49.3 (49.1–49.4)	53.9 (53.8–54.1)	53.5 (53.3–53.7)	59.1 (58.9–59.3)	< 0.001
**SES Mean (SD)**	9.2 (9.1–9.2)	8.5 (8.55–8.6)	9.4 (9.3–9.4)	8.9 (8. 9–9.0)	9.4 (9.4–9.4)	< 0.001
**Female N (%)**	119,968 (57.6%)	28,430 (58%)	26,508 (57.8%)	15,051 (57.8%)	49,979 (57.1%)	=0.083

We found that 48,873 (23.5%) of the patients were low adherers before the intervention and low adherers after it (low to low), while 87,524 (42.0%) were high adherers before the intervention and high adherers after it (high to high). Only 71,889 (34.5%) changed their adherence level after the intervention, and of these, only 45,855 (63.9%) showed improved adherence rates.

The “high to high” adherents were significantly older than the “low to low” adherents (mean age at baseline 59.1 years (CI 95%, 58.9–59.3) vs. 49.3 years (CI 95%, 49.1–49.4) [*p* < 0.05]. Among the “low to high” and “high to low” there was no difference in average age (53.9 years (CI 95%, 53.8–54.1) vs. 53.5 years (CI 95%, 53.3–53.7) (*p* = 0.684) but it was significantly higher (*p* < 0.001) than in the “low to low” group and significantly lower than the “high to high” group (*p* < 0.001).

The socio-economic level was significantly higher in the “high to high” group than in the “low to low” group (mean SES 9.4 (CI 95%, 9.4–9.4) vs. 8.5 (CI 95%, 8.55–8.6), [*p* < 0.001]. In the groups whose adherence changed, there was a significantly higher socio-economic level in those who improved their adherence (mean SES 9.4 (CI 95%, 9.3–9.4) in the “low to high” group vs. 8.9 (CI 95%, 8.9–9.0) in the “high to low” group, [*p* < 0.001].

## Discussion

We found that about 55% of patients were high adherers to a personal physician even before the policy was changed. In the year following the intervention, there was a 9.5% increase in high adherence. About one-third of our patients changed their adherence level after the intervention; 12.5% of them to a lower level.

Younger patients were more likely to show less adherence before the intervention but also afterward. As age increased, so did adherence after the intervention. Patients of low socio-economic status were less likely to be high adherers before and after the intervention. And as socio-economic status was higher, so was the increase in adherence after the intervention. Thus, the intervention did better in older patients and patients from higher SES.

The policy change in Leumit was marketed among HMO members as relevant for patients and primary care clinic staff. The change was accompanied by an organizational focus on the improvement in adherence rates. The finding that certain groups of patients will improve their adherence after such intervention needs to be further investigated to assure that the improvements achieved are long-lasting.

Surprisingly, our study shows that in a system where patients are not allocated to a personal primary care physician (i.e., as before the change), adherence rates were similar to those seen in organizations with limited patient choices for many years [7, 18, 19, 31].

Health organizations over the world invest resources in developing mechanisms to improve adherence to a personal physician. Our results raise the question of whether the adherence level is influenced by a preliminary choice of the patient and whether an organizational policy has a uniform effect on adherence.

The finding that in higher socio-economic groups, the intervention had a more positive effect on adherence may give direction for the needed intervention. For example, knowing that health literacy is better in higher socio-economic groups [33, 34] can direct health organizations to investigate further the benefits of increasing health literacy among its members, especially in those of low health literacy [ 35].

The question is, does improved adherence improve outcomes and justify the financial costs and organizational efforts? To answer this question, it is necessary to examine the cause-and-effect relationship between adherence and significant health outcomes. As mentioned before, published studies have treated patient populations as naïve ones reacting to change around them [ 9, 10]. In this study, we show that in real life, only a small but substantial part of the population is influenced, particularly those who are expected, according to their background, to have better adherence levels. It can be hypothesized that a tendency toward high adherence is a characteristic of specific patient populations. The finding that older patients show higher adherence to a personal physician and react better to the intervention can be connected to their evolving clinical situation and the need for a better relationship with a personal physician.

The major strengths of the study are its quasi-experimental design, its inclusion of the whole eligible population of a health maintenance organization, and its examination of age, sex, and SES, which are key determinants of health and health care use. Study limitations include lack of information on the number of visits or reasons for visits, reasons for continuity or lack of continuity, and additional population characteristics such as geography, urban/rural location, ethnicity, religion, language, immigration status, and health status.

The issue of the influence of health organizations on adherence is more than ever relevant. We see a decrease in care continuity [3, 10] which is not unexpected in an era where patients are accustomed to the high accessibility of services on the one hand and freedom of choice on the other [6, 13, 36]. Changes in physicians’ working conditions, partly due to regulations limiting working hours and partly due to increased numbers of private clinics where the physician can schedule service, influence service availability and the continuity of care [37, 38].

Nowadays, there is also a need to examine the relevance of defining continuity of care in terms of adherence to the same primary physician. Saltz [2] defines various types of continuity that affect primary care. These include continuity of information involving all healthcare sectors, chronological and geographic continuity of care, and especially of primary care, taking into account the patient’s family and surroundings. Nowadays, it is possible to keep information continuity without adhering to the same physician due to central electronic health records and information shared between hospitals and community clinics. Newer research should show if, under these circumstances, “continuity of information” may decrease the gaps that lower adherence may raise [39].

## Conclusion

We had a unique opportunity to present data on an organizational change of primary care services that increased adherence to the personal family physician. This policy change managed to improve adherence only in specific groups. Health organizations should direct their investments according to the potential for change and the groups in which they will want to achieve the change. They should consider interventions that improve adherence or improve health literacy in the targeted population.

Further studies are needed to evaluate the effect on clinically meaningful outcomes and patient satisfaction.

## Data Availability

The datasets analyzed during the current study are not publicly available because it is a business Information, but are available from the corresponding author upon reasonable request.

## Abbreviations

UPC: Usual provider of care
SES: Socio-economic status
